# Transcranial magnetic stimulation and preparation of visually-guided reaching movements

**DOI:** 10.3389/fneng.2012.00018

**Published:** 2012-08-08

**Authors:** Pierpaolo Busan, Marco Zanon, Federica Vinciati, Fabrizio Monti, Gilberto Pizzolato, Piero P. Battaglini

**Affiliations:** ^1^BRAIN, Center for Neuroscience, Department of Life Sciences, University of TriesteTrieste, Italy; ^2^Department of Medical and Biological Sciences, University of UdineUdine, Italy; ^3^Department of Medical, Surgical and Health Sciences, University of TriesteTrieste, Italy

**Keywords:** movement execution, parietal cortex, premotor cortex, reaching, transcranial magnetic stimulation, TMS/EEG co-registration

## Abstract

To better define the neural networks related to preparation of reaching, we applied transcranial magnetic stimulation (TMS) to the lateral parietal and frontal cortex. TMS did not evoke effects closely related to preparation of reaching, suggesting that neural networks already identified by our group are not larger than previously thought. We also replicated previous TMS/EEG data by applying TMS to the parietal cortex: new analyses were performed to better support reliability of already reported findings (Zanon et al., 2010; *Brain Topography* 22, 307–317). We showed the existence of neural circuits ranging from posterior to frontal regions of the brain after the stimulation of parietal cortex, supporting the idea of strong connections among these areas and suggesting their possible temporal dynamic. Connection with ventral stream was confirmed. The present work helps to define those areas which are involved in preparation of natural reaching in humans. They correspond to parieto-occipital, parietal and premotor medial regions of the left hemisphere, i.e., the contralateral one with respect to the moving hand, as suggested by previous studies. Behavioral data support the existence of a discrete stream involved in reaching. Besides the serial flow of activation from posterior to anterior direction, a parallel elaboration of information among parietal and premotor areas seems also to exist. Present cortico-cortical interactions (TMS/EEG experiment) show propagation of activity to frontal, temporal, parietal and more posterior regions, exhibiting distributed communication among various areas in the brain. The neural system highlighted by TMS/EEG experiments is wider with respect to the one disclosed by the TMS behavioral approach. Further studies are needed to unravel this paucity of overlap. Moreover, the understanding of these mechanisms is crucial for the comprehension of response inhibition and changes in prepared actions, which are common behaviors in everyday life.

## Introduction

Several works have tapped on neural underpinnings of reaching movement preparation, focusing on parieto-frontal circuits (e.g., Andersen and Buneo, 2002; Andersen and Cui, 2009; Cisek and Kalaska, 2010). In particular, reaching movements under visual guidance are prepared using different information which is elaborated in different frames of reference. These are, for example, eye-, limb-, body- or head-centered (Cohen and Andersen, 2002; Beurze et al., 2010) using visual as well as proprioceptive information (Buneo et al., 2002; Filimon et al., 2009; Jackson et al., 2009). Since preparation of reaching movements involves the activation of a fronto-parietal network (Tannè et al., 1995; Johnson et al., 1996; Galletti et al., 2001; Marconi et al., 2001; Gamberini et al., 2009; Bakola et al., 2010; Passarelli et al., 2011), it has been hypothesized that it integrates information about physical properties and location of a target into the motor plan of a reaching movement (Buneo et al., 2002; Cohen and Andersen, 2002). In fact, Milner and Goodale (2006) suggested the existence of a dorsal stream that mediates sensory-motor transformations for visually-guided movements overlapping with the above mentioned anatomic regions. They also suggested the existence of a ventral stream, which would be more involved in the elaboration of object's features primarily involving occipito-temporal regions.

An unsolved issue regarding implementation of reaching movements is related to the possible dominance of one hemisphere, preferably the left one (Goodale, 1988), usually viewed as the dominant hemisphere in right-handed people (e.g., Iacoboni, 2006; Vingerhoets et al., 2011). Thus, the left hemisphere likely plays a special role in organizing movements during visually-guided reaching (Goodale, 1988). The contralateral limb may be more represented during planning of reaching, activating a wide series of neural networks (e.g., Kertzman et al., 1997; Medendorp et al., 2003, 2005). The activation of both hemispheres in similar tasks (Calton et al., 2002; Connolly et al., 2003; Prado et al., 2005) or of structures modulated by ipsilateral reaching has also been reported (Chang et al., 2008; Busan et al., 2009b). However, in our previous and present investigations, we mainly concentrated on the left hemisphere of subjects using their right hand, considering that this should allow to individuate the better representation of neural circuits for reaching (see above).

By stimulating medial parieto-occipital, parietal and premotor regions with TMS, we have previously identified a discrete network of regions that were involved in the preparation of reaching movements (Busan et al., 2009a,c). Specifically, at the start of preparation, we induced a facilitation in reaction time (RT) in a medial parieto-occipital region near the parieto-occipital sulcus, independently of the use of foveal or peripheral vision, and independently of the target position (however, strongest effects were observed for foveal vision and central reaching). Moreover, the stimulation of a region close to the posterior parietal cortex resulted in slower RT when TMS was delivered at about half of the preparatory process, affecting only central reaching. This same region was facilitated (showing faster reaction times) when stimulating at the start of reaching preparation. This was explained by the state-dependent theory of TMS (Silvanto and Muggleton, 2008): the TMS effect may strongly depend on the excitability of the stimulated region. Referring to our data, TMS could “pre-activate” cortical regions at the start of the preparatory process before their effective involvement in the stream, facilitating their intervention. In contrast, when cortical regions are already involved in the task, the adjunction of “neural noise” (Miniussi et al., 2010) may interfere with their correct functioning and, consequently, a slower elaboration of information may result.

When stimulating at about half of the mean RT in a more anterior left parietal region (around the intraparietal sulcus), we were able to induce an additional shortening of RT, facilitating the preparation of reaching (Busan et al., 2009a). Moreover, we were able to evoke a similar effect stimulating the left premotor dorsal cortex, in the same time window, suggesting a parallel processing of information in these cortical regions (Busan et al., 2009a).

We have now extended the mapping of cortical areas possibly involved in preparing visually-guided reaching movements. We tested whether the application of TMS to regions more laterally located in comparison to previous ones will affect the reaching movement preparation. Occipital, parietal and premotor cortices were stimulated. Negative results would have implied that the preparation of natural reaching is strictly related to structures in the superior parietal lobule (SPL). This should support the hypotheses of a “dorso-medial” stream that is preferentially involved in reaching movements, classically opposed to a “dorso-lateral” stream, possibly more devoted to grasping and/or reach-to-grasp movements (Jeannerod et al., 1995; Davare et al., 2006, 2010; Koch et al., 2010). On the contrary, if TMS would have elicited any effect, this would suggest a wider extent of the stream and a role for some of its more lateral regions in the preparation of reaching (Koch et al., 2008; Vesia et al., 2008, 2010; Reichenbach et al., 2011).

We also were interested in understanding the relations among cortical regions possibly involved in the preparation of reaching. Using a TMS/Electroencephalography (EEG) co-registration approach, we individuated an ensemble of areas recruited by stimulation of the left parietal cortex (putatively around the intraparietal sulcus) that comprises areas of the temporo-occipital regions (i.e., the ventral stream; Zanon et al., 2010). This confirms an interchange of information between dorsal and ventral streams, and that they are not segregated systems (e.g., Schenk and Milner, 2006; Borra et al., 2008), adding information about the temporal dynamics of this activity.

We also wished to further characterize the temporal dynamics of activation elicited by stimulation of the previously investigated region (Zanon et al., 2010), thus we used a TMS/EEG co-registration approach replicating that experiment, but adopting different analyses. Slightly different, but compatible findings were expected that would strengthen the previous conclusions.

A better understanding of the circuitry involved in the preparation of reaching is important for practical purposes such as, for example, the implementation of rehabilitative protocols or prosthetic devices. Moreover, the understanding of the organization and physiology of response inhibition could be helped by the knowledge of the physiology and organization of unrestrained reaching movements.

## Materials and methods

### Behavioral TMS study

#### Subjects

A total of 58 healthy subjects underwent TMS over different cortical regions, as reported in Table 1. Subjects were right-handed (Edinburgh Inventory; Oldfield, 1971). Participants gave written informed consent after receiving information about TMS, in compliance with the Declaration of Helsinki and the Ethics Committee of the University of Trieste. Participants could leave the study at any time, although all completed the experiments. These statements apply also to TMS/EEG procedures.

**Table 1:** **Stimulated areas and subjects recruited for the behavioral experiments**.

**Stimulated cortical region (left hemisphere)**	**Time of stimulation (% of m-RT)**	**Subjects**			
		***N***	**males/females**	**Age range**	**Mean age/standard deviation**
Lateral parieto-occipital cortex	25	13	8/5	20–29	23.4/3.0
Lateral parieto-occipital cortex and lateral premotor cortex	50 and 75	10	6/4	20–27	24.8/3.1
Inferior parietal lobule	25	9	5/4	20–30	23.9/2.8
Inferior parietal lobule	50	10	3/7	22–31	25.5/3.1
Inferior parietal lobule	75	16	9/7	22–46	25.5/3.5
Lateral premotor cortex	25	10	6/4	20–27	24.8/3.1

The time of stimulation with respect to the onset of movements is also reported. The same 10 subjects participated in premotor cortex and parieto-occipital (with TMS at 50% and 75% of m-RT) experiments.

#### Cortical stimulation

TMS was delivered over three brain regions of the left hemisphere: five scalp locations were in parietal cortex, four in premotor cortex, and one in parieto-occipital cortex. For each location, TMS was delivered at three different times during preparation of reaching: 25% of mean reaction time (m-RT), 50% of m-RT, and 75% of m-RT (Table 1).

TMS (Medtronic MagPro R30) was delivered through a figure-of-eight coil (diameter of each wing about 7 cm), oriented tangentially to the scalp (single pulse stimulation; biphasic waves; pulse duration: 280 μs). The coil was secured on the scalp by hand and position was checked and readjusted if necessary. The coil was maintained with a 45° orientation with respect to the inter-hemispheric fissure with the handle pointing downward and backward.

Subject's heads were not restrained, although participants were asked to maintain a stable position for the entire experiment. The stimulation coil was maintained in position even when no TMS was delivered.

**Figure 1:**
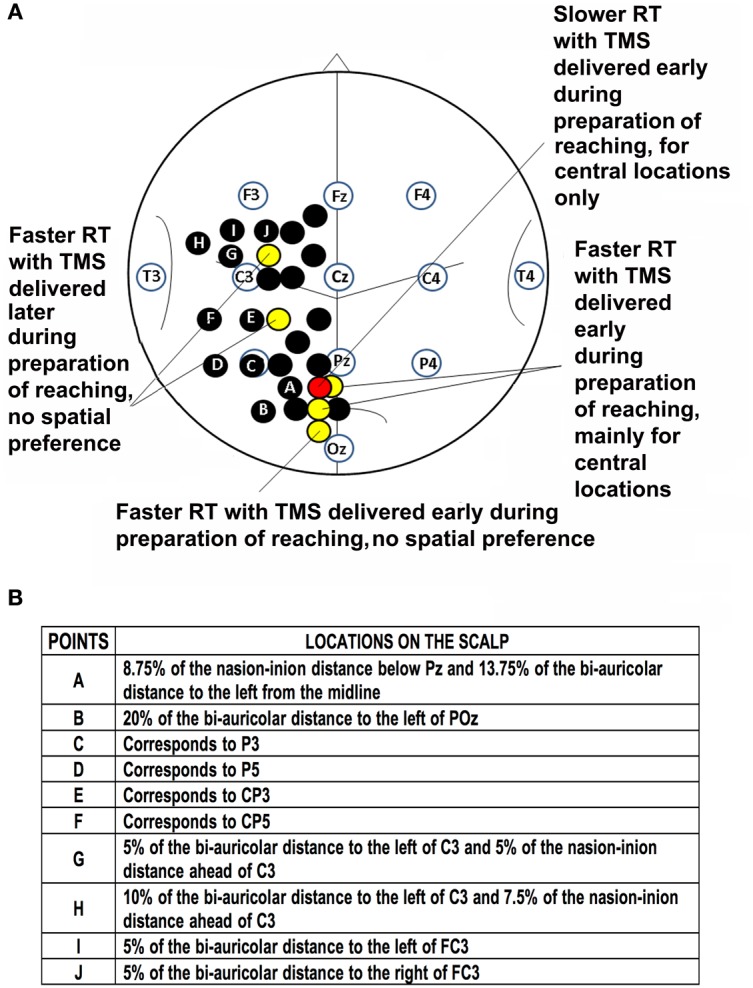
**Location of points of stimulation on the scalp and previous results. (A)** Schematic drawing of the scalp showing points of stimulation in previous investigations (black, yellow and red circles; Busan et al., 2009a,c) and the present work (black circles with letters). Positions of selected scalp EEG electrodes are also reported together with an outline of main sulci. Point A corresponds to a parieto-occipital lateral position, points B-F are positioned over the putative inferior parietal lobule, while points G-J are positioned over the putative premotor cortex. (**B**) Table of stimulated points and related locations on the scalp.

Stimulated scalp positions, were determined according to an adapted EEG coordinate system (e.g., Herwig et al., 2003; Jurcak et al., 2007) and using a probabilistic method (Steinsträter et al., 2002; http://www.neuro03.uni-muenster.de/ger/t2tconv/). Points of stimulation were marked on a cap. Stimulated points with the underlying main sulci are shown in Figure 1. In each experiment, scalp locations were randomly stimulated in blocks.

#### Pre-experimental procedures

Before each experiment, the best point activating the right hand first dorsal interosseous muscle (FDI) was determined and the resting motor threshold (RMT) was the stimulus intensity triggering at least a 50 μV response on electromyography (EMG; band pass filtering 20–2000 Hz) in half of stimulations. Surface Ag/AgCl electrodes were used (tendon-belly montage). Intensity of TMS was then set at 120% RMT. When stimulating premotor areas, the intensity of TMS was 110% RMT to limit current diffusion to primary motor cortex. Before the experiment, stimulation points in premotor cortex were evaluated for the possibility that stimulation led to muscular responses on hand/tongue muscles by EMG. When a muscular response was evident, the point of stimulation was slightly moved anteriorly until muscular response was no longer evident. Clearly, these procedures could make the individuation of premotor cortex uncertain, but we preferred to minimize contamination from neighboring neural structures, such as the motor cortex.

#### Experimental setting

During experiments, each subject was seated at a table. He/she was asked to place his/her right hand on a sensor light placed 5 cm away from his/her chest. The sensor light was connected to an impedance detector allowing measurements of the RT (the time elapsed from the go-signal to movement onset). A metallic grid was placed over the table, covering three light emitting diodes (LEDs) positioned at the center, right, and left at about 35 cm from subjects. LEDs were covered by a white sheet and were visible when illuminated only. LEDs to the right and left of subjects were positioned at 40° from the central one. LED lighting was the go-signal and the subject was asked to move the hand from the sensor, toward the grid to reach the LED. A cross drawn along the midline of the grid was used to maintain steady fixation during the experiment. Subject reached targets on the left and the right using peripheral vision, while the central target was reached using foveal vision. This requirement forced the inhibition of saccadic movements toward the target when it was lateral. However, this was necessary to investigate the effect of peripheral vision in the preparation of reaching. Arm and eye movements were recorded with a digital video camera (Sony DCR-SR30E) to discard incorrect trials. Timing of TMS delivery and all events were controlled by a PCMCIA acquisition board (NI-DAQ 6024E, National Instruments, Texas, USA) allowing RT recording. Before experiments, subjects performed about 20 reaching trials with targets distributed to the center, right, and left to measure their m-RT.

At the beginning of each trial, subjects focused their attention on the cross. They maintained their right hand on the light sensor before the LED was lit. This signaled the subject to move as soon as possible, maintaining steady central fixation. Subjects performed 42 randomized trials for each stimulated point: 21 trials with and 21 without TMS (TMS and NO-TMS conditions; 14 trials for every target location, 7 with and 7 without TMS) for each point and for each TMS condition (25%, 50%, 75% of m-RT). If, after the execution of a block, the m-RT was reduced more than 20% (due to implicit learning or familiarization with apparatus), a new m-RT was measured considering the last NO-TMS trials.

#### Data treatment and statistical analysis

TV recordings were analyzed off-line, excluding trials where eyes did not remain on the central cross for the entire trial duration. To avoid the influence of inadequate attention, trials with an RT longer than 700 msec or shorter than 100 msec were excluded. Trials were discarded when trajectory corrections were made. Data that were beyond two standard deviations with respect to the mean of the condition were discarded. Statistical analysis on RT was conducted with repeated measures ANOVA (see the “Results” section). A *p* < 0.05 was considered as the significant threshold. When interactions among main effects were significant, further analyses were conducted to explore these effects (see the “Results” section). The normality of data was also checked.

#### Control experiments

Past and present experiments detected different effects due to methodological issues, also in relation to the TMS state-dependent theory (Silvanto and Muggleton, 2008). Thus, we replicated part of previous experiments (see Busan et al., 2009c) in which a slightly different setting was used. In those experiments, subjects reached a solid target: the go-signal was given with eyes closed, so as not to see the positioning of the target itself. Thus, a “double” RT was registered, composed of the time needed to open the eyes and that required to start movements. The stimulation of a region over medial SPL (5% of the nasion-inion distance below Pz and 5% of the bi-auricular distance to the left) was effective in increasing RT at 75% of the (double) m-RT.

In eight right-handed, healthy subjects (three males, five females; age range 20–25, mean age 22.4, standard deviation –SD- 1.5), we performed the same experiment as the previous by applying TMS at 75% of m-RT of the (double) RT over medial SPL.

Results were compared to those collected in a second control experiment, where six right-handed, healthy subjects (two males, four females; age range 22–29; mean age 23.7, SD 2.7) performed the same task, but with open eyes and using LEDs instead of the solid target. TMS was applied at 50% of m-RT, a time considered as equivalent to the 75% of the (double) m-RT in previous experiments. Statistical comparisons were made using Student's *t*-test. The results obtained from these settings have been qualitatively compared to evaluate the possibility that different effects could be observed with respect to slightly different experimental requests, possibly sustaining the TMS state-dependent theory (Silvanto and Muggleton, 2008).

#### TMS/EEG study

A TMS/EEG experiment was carried out to confirm and extend previous findings (Zanon et al., 2010). Nine right-handed healthy subjects (five males and four females, age range 20–26 years, mean age 23.9 years, SD 2.1) participated in these experiments. Subjects were seated with closed eyes for the duration of blocks to reduce ocular artifacts.

TMS apparatus, coil orientation, protocols, instrumentations and data acquisition procedures were the same as those described in Zanon et al. (2010). Stimuli were delivered on the left parietal cortex on the same scalp location stimulated previously (Zanon et al., 2010). It corresponded to a region putatively involved in reaching preparation (Busan et al., 2009a).

#### EEG and data analysis

EEG traces were recorded and treated as in Zanon et al. (2010). For data analysis, an average of 95.7 (SD 15.3) epochs was considered for real TMS and 89.7 (SD 15.3) for sham. Part of the TMS artifact as well as all the other remaining artifacts were eliminated as much as possible using EEGLAB (Delorme and Makeig, 2004). The first 20 msec prior to the delivery of stimulations and 35 msec after them were deleted. Independent component analysis (Jung et al., 2000) allowed for elimination of artifacts (e.g., those related to the slow decay/recovery after TMS).

Epochs were then averaged to obtain real and sham TMS evoked potentials (TEPs). Considering the remaining TMS artifact that could influence the analyses, a “linear detrend” function was applied when needed, generally in a time between 35 and 300 msec after the delivery of stimuli. Finally, averaged real and sham TEPs were re-referenced (average reference).

sLORETA (standardized low resolution brain electromagnetic tomography; Pascual-Marqui, 2002) was used to compute the cortical three-dimensional distribution of neuronal activity comparing real and sham TEPs. sLORETA is a standardized discrete, three-dimensional distributed, linear, minimum norm inverse solution (Pascual-Marqui, 2002). Computations were made in a realistic head model (Fuchs et al., 2002) using the MNI152 template (Mazziotta et al., 2001), with three-dimensional space solution restricted to cortical gray matter, as determined by the probabilistic Talairach atlas (Lancaster et al., 2000), and with electrode positions superimposed on the MNI152 scalp (Oostenveld and Praamstra, 2001; Jurcak et al., 2007). The intracerebral volume is partitioned in 6239 voxels at 5 mm spatial resolution. Anatomical labels such as Brodmann areas are also reported using MNI space, with corrections to Talairach space (Brett et al., 2002).

In the present work, sLORETA was used to perform a voxel-by-voxel within-subjects comparison of real vs. sham TMS induced current density distribution in the brain. Significant differences in EEG source maps were assessed with non-parametric statistical analysis (Statistical non-Parametric Mapping: SnPM; Nichols and Holmes, 2002), as previously described (Zanon et al., 2010).

We reduced the localization error by applying regularization in the source reconstruction. We considered the mean signal-to-noise ratio of averaged ERPs, for each subject in every condition, from 35 to 300 msec after delivery of the stimulus with respect to baseline.

After reconstructing the EEG cortical sources distribution for both real and sham TMS conditions, analyses were conducted considering single time frames in a time ranging from 35 to 300 msec after stimulation. Statistics were implemented also considering the mean neural activity in time windows individuated by visual inspection of TEPs. The following comparisons were implemented: from 35 to 60 msec, from 60 to 130 msec, from 130 to 245 msec, and from 245 to 300 msec. Significance was set at *p* < 0.05, correcting for multiple comparisons. SnPM in sLORETA allowed for correction of multiple comparisons even with respect to all voxels and all time samples.

## Results

### Behavioral TMS study

We considered the main effects and interactions among TMS (yes/no), location of stimulation on the scalp (one, four, or five positions), target position in space (central, left, and right) with repeated measures ANOVA. We conducted analyses for each timing of TMS delivery. Student's *t*-test (Bonferroni corrected) was used to characterize significant findings. When a three-way interaction was significant, the statistical model was investigated by two-way interactions and then with a Student's t-test (Bonferroni corrected). Results obtained for all conditions (Figure 1) are summarized in Table 2. We observed that an effect related to target position was always evident (parieto-occipital cortex: 25% m-RT: *p* = 0.043; 50% m-RT: *p* = 0.012; 75% m-RT: *p* = 0.013; parietal cortex: always *p* < 0.009; premotor cortex: always *p* < 0.009), mainly indicating that subjects had longer RTs for movements toward the left targets. Moreover, TMS always resulted in faster RT when stimulating at 25% of m-RT (TMS main factor, independently of target position, always *p* < 0.009).

**Table 2:** **Reaction times observed in behavioral experiments**.

**Points**	**Target location**	**25% of m-RT**		**50% of m-RT**		**75% of m-RT**	
		**TMS**	**NO-TMS**	**TMS**	**NO-TMS**	**TMS**	**NO-TMS**
A	Central	287.6 (39.6)	301.1 (39.6)	286.6 (24.9)	293.2 (25.9)	296.0 (32.6)	290.3 (29.3)
	Left	292.9 (47.9)	318.6 (43.0)	298.8 (25.0)	305.2 (30.3)	303.1 (22.2)	309.5 (27.6)
	Right	284.5 (34.1)	301.7 (39.8)	288.1 (31.5)	287.2 (30.4)	292.2 (23.8)	290.7 (22.8)
B	Central	290.2 (39.5)	304.3 (40.7)	348.4 (64.3)	345.0 (79.8)	309.6 (35.9)	310.8 (34.9)
	Left	327.0 (56.6)	341.4 (59.1)	382.2 (69.7)	391.1 (68.3)	333.5 (51.4)	336.6 (49.4)
	Right	284.8 (38.7)	296.4 (41.3)	328.9 (58.2)	339.2 (67.9)	299.9 (39.4)	302.9 (38.9)
C	Central	298.2 (46.4)	309.2 (49.2)	338.1 (54.1)	342.4 (58.4)	308.9 (40.6)	308.0 (39.6)
	Left	314.4 (62.2)	346.6 (66.1)	380.7 (61.7)	374.1 (63.3)	338.1 (60.6)	334.4 (60.4)
	Right	279.8 (36.5)	294.7 (42.1)	323.9 (40.4)	330.1 (49.5)	306.4 (40.9)	302.7 (43.5)
D	Central	290.9 (42.0)	308.7 (43.5)	349.0 (56.6)	351.4 (52.6)	314.7 (45.2)	311.7 (46.7)
	Left	308.5 (66.6)	333.5 (68.9)	389.0 (71.6)	387.7 (65.2)	340.4 (58.5)	343.7 (62.3)
	Right	287.9 (44.5)	299.5 (37.3)	329.4 (53.5)	339.3 (52.7)	311.2 (44.5)	312.9 (40.3)
E	Central	292.4 (42.3)	305.5 (43.7)	348.2 (49.9)	342.2 (57.0)	316.5 (45.9)	324.7 (52.4)
	Left	323.4 (72.7)	346.5 (63.8)	389.2 (61.1)	383.6 (64.0)	344.4 (62.9)	350.3 (62.6)
	Right	277.0 (38.2)	298.2 (40.3)	327.5 (44.7)	329.9 (49.3)	308.4 (43.5)	307.7 (41.3)
F	Central	292.3 (46.9)	305.8 (42.6)	359.9 (61.7)	369.1 (58.8)	312.2 (44.2)	313.0 (40.7)
	Left	321.3 (62.7)	338.8 (65.2)	395.6 (72.2)	400.2 (81.6)	347.3 (59.6)	341.7 (62.0)
	Right	287.3 (39.8)	294.7 (38.4)	340.7 (56.9)	352.2 (59.3)	300.1 (36.8)	305.7 (37.5)
G	Central	267.3 (22.4)	293.8 (21.7)	289.3 (22.6)	291.5 (23.0)	292.5 (27.7)	291.6 (35.6)
	Left	273.7 (12.4)	305.6 (26.2)	305.2 (21.1)	307.4 (28.9)	310.0 (25.6)	303.4 (34.4)
	Right	267.8 (21.9)	297.4 (20.1)	282.5 (20.2)	292.3 (29.9)	292.7 (22.1)	287.9 (29.7)
H	Central	270.8 (21.1)	285.7 (24.5)	296.7 (17.4)	289.6 (21.1)	283.4 (21.2)	286.8 (31.1)
	Left	288.7 (27.1)	303.1 (27.8)	301.9 (15.8)	313.8 (27.8)	297.9 (22.7)	300.6 (26.1)
	Right	275.8 (25.5)	286.0 (27.2)	278.7 (23.6)	286.2 (23.5)	283.8 (20.4)	285.9 (24.7)
I	Central	278.9 (16.7)	297.1 (18.2)	292.2 (27.4)	293.7 (25.9)	290.8 (20.2)	288.5 (26.4)
	Left	287.1 (22.1)	304.3 (25.9)	296.4 (29.8)	306.8 (32.7)	300.3 (30.5)	303.7 (26.7)
	Right	278.9 (25.8)	299.5 (26.5)	289.6 (27.8)	287.1 (25.2)	290.9 (22.4)	290.5 (29.1)
J	Central	272.2 (22.3)	294.5 (21.3)	288.7 (18.7)	292.1 (21.4)	288.3 (25.7)	286.3 (33.0)
	Left	285.7 (30.6)	313.6 (25.7)	309.8 (30.1)	312.8 (23.0)	309.0 (28.4)	301.1 (27.0)
	Right	267.9 (21.4)	293.1 (30.4)	287.3 (27.0)	293.4 (26.8)	292.9 (26.4)	284.2 (24.6)

Mean of reaction times (SD in brackets) obtained for parieto-occipital (point A), parietal (points B–F), and premotor (points G–J) cortices.

When considering the premotor cortex, we observed an interaction of TMS vs. location of stimulation at 25% of m-RT (*p* = 0.004). However, subsequent analyses showed the presence of significant effects on all stimulated points (point G: *p* < 0.009; point H: *p* < 0.009; point I: *p* < 0.009; point J: *p* < 0.009 in Figure 1). Due to the wide distribution of effective points of stimulation, we considered these results as unspecific effects, likely confirming that the facilitating effect caused by TMS when stimulating at 25% of m-RT was not related to a genuine effect (e.g., Sawaki et al., 1999). All remaining comparisons never reached significance.

### Control experiments

Two control experiments were performed. In the first, we replicated the original result of slowed RT (TMS mean reaction time: 690.25, SD 101.5; no-TMS mean reaction time: 652.8, SD 109.2; *p* = 0.04) when stimulating medial left posterior parietal cortex using the original experimental setting (Busan et al., 2009c), reaching toward the center. We performed a second experiment where subjects were required to keep their eyes open. In this instance we did not replicate findings of the previous experiment, and no significant differences were evident between TMS and no-TMS when reaching toward the center with foveal observation (TMS mean reaction time: 309.0, SD 33.8; no-TMS mean reaction time: 312.1, SD 30.0; *p* = 0.60).

### TMS/EEG study

#### ERP description and sLORETA

**Figure 2:**
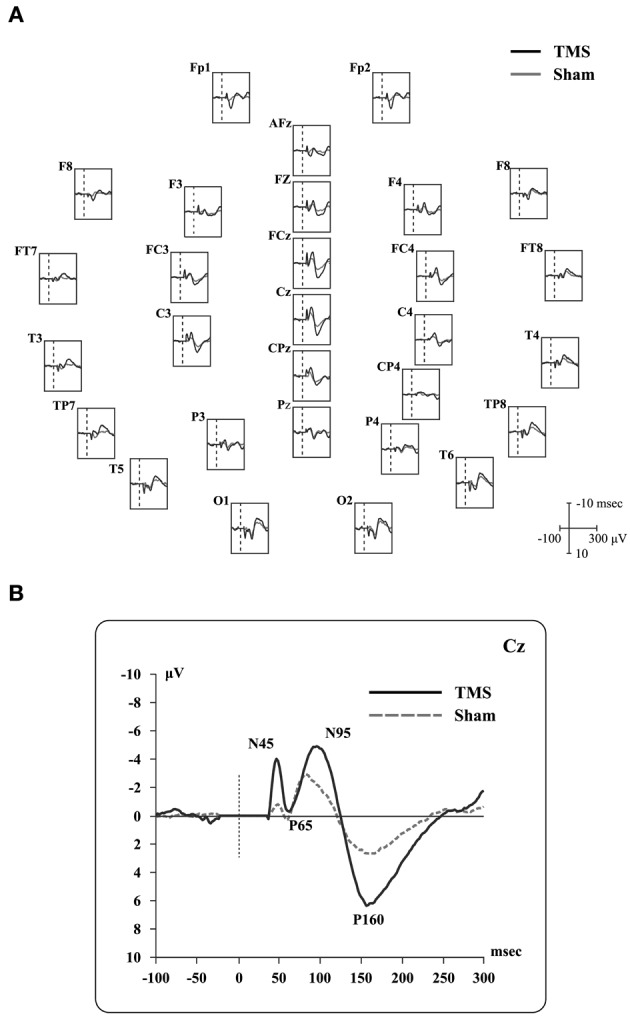
**Real TMS and sham TMS evoked potentials in 9 healthy subjects.** (**A**) Grand-average of real TMS evoked potentials ranging from 100 msec before to 300 msec after the stimulation, showing all recorded electrodes. Real TMS evoked potentials are superimposed on sham-evoked potentials. Site of stimulation over the parietal cortex is also shown. (**B**) Grand-average of evoked potentials recorded at the Cz electrode, showing the main deflections described in the text.

Real and sham TEPs showed positive and negative deflections (see, for example, Paus et al., 2001; Bonato et al., 2006). Specifically, when Cz electrode was considered, we observed four peaks (Figure 2) during real TMS: (i) a negative component (N45; mean amplitude −4.2 microvolts, SD 3.0; mean latency 44.8 msec, SD 2.4), (ii) a positive one (P65; mean amplitude 1.7 microvolts, SD 1.5; mean latency 63.2 msec, SD 5.2), followed by (iii) a negative one (N95; mean amplitude −6.6 microvolts, SD 2.1; mean latency 95.5 msec, SD 11.7), and, finally, (iv) a positive one (P165; mean amplitude 7.2 microvolts, SD 2.3; mean latency 161.9 msec, SD 15.3). Sham TEPs showed similar deflections with reduced amplitudes compared to real TMS (Figure 2). The acoustic contamination was evident (Nikouline et al., 1999) in both conditions.

Time frame by time frame comparison of the entire time window showed a significant (*p* < 0.05) difference between the real and sham TMS in the time ranges between 116–126 msec, 134–146 msec, and at about 190 msec after the delivery of stimuli.

In the first time range (116–126 msec), we observed significant voxels in the left postcentral gyrus, left inferior parietal lobule (IPL), and right motor regions. In the same interval, but with a slight delay, further significant voxels were also evident in the right cuneus, right middle occipital gyrus, right lingual gyrus, right precuneus, right SPL, right IPL, as well as in the right angular gyrus. The cingulate gyrus and the posterior cingulate also showed significant voxels in this time window.

In the second time range (134–146 msec) we observed significant voxels in the right SPL, right IPL, right supramarginal gyrus, right postcentral gyrus, precuneus and sub-gyral. Finally, at 190 msec after TMS we observed significant voxels in the left superior temporal gyrus and supramarginal gyrus.

**Figure 3:**
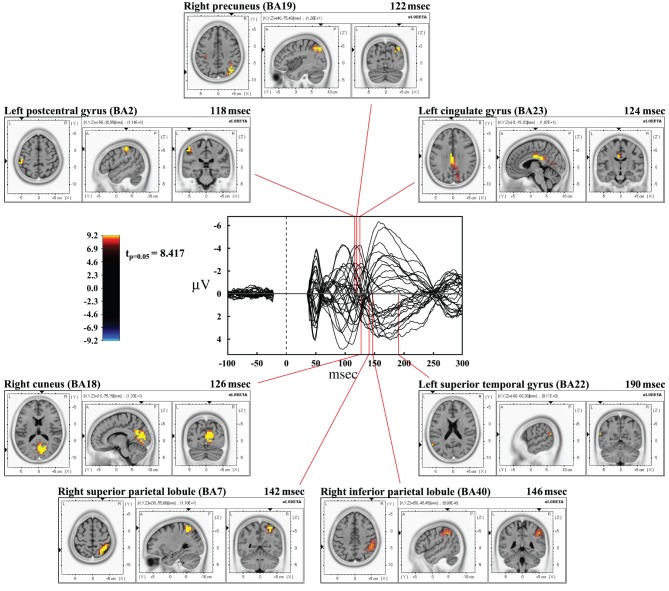
**Results from time frame by time frame sLORETA analysis.** Representation of the main patterns of activation obtained in time frame by time frame analysis compared to a butterfly plot showing the grand-averaged (for all subjects) evoked potentials (real TMS) for each electrode.

Maximal peaks of activation and number of voxels activated for each significant time frame are reported in Table 3. The main patterns of activations are shown in Figure 3.

**Table 3:** **Results from time frame by time frame sLORETA analysis**.

**Time of activation (msec)**	**Maximal peak of activation**			**Other significant voxels (BA)**	**Number of activated voxels**
	***x, y, z* (MNI coordinates)**	**BA**	**Anatomical landmark**		
116	−50, −30, 55	2	Left postcentral gyrus	40 L, 1 L, 4 R	15
118	−45, 25, 50	2	Left postcentral gyrus	1 L, 3 L, 19 R	10
120	40, −75, 40	19	Right Precuneus	2 L, 3 L, 7 R, 18 R, 30 R, 31 R, 39 R, 40 L/R	57
122	40, −75, 45	7	Right superior parietal lobule	18 R, 19 R, 23 L/R, 31 R, 39 R	45
124	−5, −15, 30	23	Left cingulate gyrus	17 R, 18 R, 19 R, 23 R, 24 L, 29 R, 30 R, 31 L/R	111
126	10, −75, 15	18	Right cuneus	7 R, 17 R, 23 R, 30 R, 31 R	109
134	20, −60, 55	7	Right precuneus	/	11
136	25, −60, 60	7	Right superior parietal lobule	5 R	42
138	30, −60, 60	7	Right superior parietal lobule	5 R, 40 R	27
140	30, −55, 60	7	Right superior parietal lobule	2 R, 5 R, 40 R	44
142	30, −60, 60	7	Right superior parietal lobule	2 R, 5 R, 40 R	66
144	35, −60, 55	7	Right superior parietal lobule	5 R, 40 R	52
146	50, −45, 45	40	Right inferior parietal lobule	/	23
190	−60, −60, 20	22	Left superior temporal gyrus	40 L	2

Time of activation and location of maximal peaks which were significant with analysis made on the entire window of interest (35–300 msec). The remaining significant voxels are also reported. BA: Brodmann Areas; L = left; R = right.

**Figure 4:**
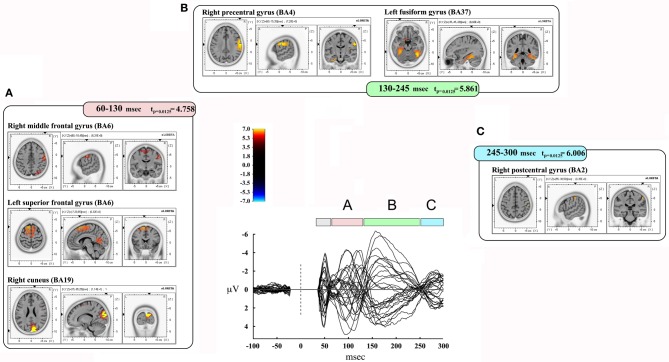
**Results from discrete time window sLORETA analysis.** Representation of some of the main results obtained when considering the mean neural activity in discrete time windows. Results are plotted compared to the different time ranges considered, as evident from the butterfly plot showing the grand-averaged (for all subjects) evoked potentials (real TMS) for each electrode. Colors and letters are plotted in correspondence to the different time windows of interest. **(A)** Time window from 60 to 130 msec. **(B)** Time window from 130 to 245 msec. **(C)** Time window from 245 to 300 msec.

We also took into consideration the mean activation across the overall four time-windows of interest (Figure 4). In this case, significance was *p* < 0.0125. The first time window (from 35 to 60 msec) did not reach the threshold for significance, while it was reached in the second time window (60–130 msec): we observed significant voxels in the contralateral posterior regions (mainly in the cuneus and precuneus –bilaterally-, IPL and SPL, the middle occipital gyrus, the lingual gyrus –bilaterally-, the cingulate gyrus, and posterior cingulate –bilaterally). Significant voxels were also evident in the right inferior, middle, and superior temporal gyrus. Finally, we observed significant voxels in the left and right superior, middle and medial frontal gyrus, right inferior frontal gyrus, left and right precentral gyrus, right postcentral gyrus, and left sub-gyral.

The third time window (130–245 msec) showed a pattern of significant voxels that comprised the right precentral and postcentral gyrus, right inferior frontal gyrus, and the left medial frontal gyrus. Posteriorly, we observed significant voxels in the right and left fusiform gyrus, left uncus, left and right parahippocampal gyrus, right cuneus and precuneus, right posterior cingulate, right lingual gyrus and right IPL. Finally, significant propagation was seen also in the left and right inferior temporal gyrus and the right insula.

The last window, ranging from 245 to 300 msec, showed activations in the right postcentral gyrus and left superior temporal gyrus. Results are summarized in Table 4 and Figure 4.

**Table 4:** **Results from discrete time windows sLORETA analysis**.

**Window of activation (msec)**	**Maximal peak of activation**			**Other significant voxels (BA)**	**Number of voxels activated**
	***x, y, z* (MNI coordinates)**	**BA**	**Anatomical landmark**		
35–60	N.S.	N.S	N.S.	N.S.	N.S.
60–130	15, −95, 25	19	Right cuneus	1 R, 2 R, 3 R, 4 R, 6 L/R, 7 R, 8 L/R, 9 R, 17 R, 18 L/R, 20 R, 21 R, 23 L/R, 29 L/R, 30 L/R, 31 L/R, 32 L, 38 R, 39 R, 40 R	513
130–245	60, −15, 35	4	Right precentral gyrus	1 R, 2 R, 3 R, 6 R, 9 R, 13 R, 17 R, 18 R, 19 L/R, 20 L/R, 23 R, 25 L, 28 L, 30 R, 31 R, 34 L, 35 L, 36 L/R, 37 L/R, 40 R	280
245–300	55, −30, 50	2	Right postcentral gyrus	22 L	4

Time of activation and location of maximal peaks which were significant are reported. The remaining significant voxels are also reported. BA: Brodmann Areas; L, left; R, right; N.S. = Not Significant.

## Discussion

In the present investigation, we report findings obtained by stimulating cortical areas along a “dorso-lateral” stream in the left hemisphere in healthy right-handed people during the preparation of visually-guided reaching movements performed with the dominant hand. Lateral parietal and premotor regions resulted not to be strictly involved in the preparation of visually-guided reaching as measured by the present protocol. This confirms that the neural network for preparation of reaching is quite localized, as already suggested by our previous works (Busan et al., 2009a,c).

The following discussion will focus on the parietal cortex, considering that our previous results on dorsal premotor cortex (Busan et al., 2009a) could be related to non-specific effects, even if we are well aware that the premotor cortex plays a role in the preparation of motor responses and in reaching (e.g., Prado et al., 2005; Pesaran et al., 2006; Batista et al., 2007; Hoshi and Tanji, 2007; Beurze et al., 2010).

### Segregated systems for the preparation of reaching movements?

The present findings suggest that the neural network herein stimulated is not strongly involved in visually-guided reaching movements. Our previous studies (Busan et al., 2009a,c) showed the presence of a discrete dorsal neural circuit starting from the medial parieto-occipital cortex, involving the SPL near the intraparietal sulcus and the dorsal premotor cortex. However it should be kept in mind that the effect of TMS could be due to the modulation of regions simply linked to the stimulated area.

The left hemisphere was chosen for its dominance for praxis (e.g., Goodale, 1988; Haaland and Harrington, 1989), and the left parietal cortex involvement in arm movement planning (Rushworth et al., 2003; Wheaton et al., 2009). SPL lesions in the left hemisphere can result in misreaching throughout the workspace (Perenin and Vighetto, 1988), while a right hemisphere lesion may be more related with lateralized visual field effects (Perenin and Vighetto, 1988; Battaglia-Mayer et al., 2006). However, left parietal lesions may have effects only in the contralateral visual field (Riddoch, 1935). Thus, the posterior parietal cortex plays a pivotal role in the preparation of actions (Goodale and Milner, 1992; Jeannerod et al., 1995; Andersen et al., 1997) with a contralateral limb bias in more anterior parietal regions and less evident in the parieto-occipital cortex (Busan et al., 2009b; Vesia et al., 2010).

Our previous and present results are in agreement with literature (e.g., Kastner et al., 1998; Fattori et al., 2001, 2005; Andersen and Buneo, 2002; Calton et al., 2002; Snyder et al., 2006; Trillenberg et al., 2007; Ciavarro and Ambrosini, 2011; Striemer et al., 2011) supporting a role for SPL in preparation of reaching (Goodale and Milner, 2004; Buneo and Andersen, 2006). In particular, Beurze et al. (2010) found parieto-occipital activations related with foveal vision comparable with our results.

Striemer et al. (2011) suggested that SPL is preferentially related to programming of actions and on-line control, while IPL should not. The latter should be more related with selecting the goal and/or target of the action. They found an effect on endpoint accuracy when using a triple pulse TMS over SPL during the preparation of movement, while this was not observed when stimulating IPL. They also observed a significant reduction in RT, which might be related to unspecific effects but it might also be a genuine result. In fact, this finding is congruent with our previous results (Busan et al., 2009a,c). Striemer et al. (2011) suggested that those results might be correlated to attentional or intentional processes rather than to motor planning. Along these lines, we already controlled the possibility that unspecific attention processes biased our results (Busan et al., 2009a,c), but the possibility that they were also related to attention processes specifically related to reaching movements cannot be ruled out. However, these processes should be more likely related with IPL than with SPL involvement (Rushworth et al., 2001; Desmurget and Sirigu, 2009). Striemer et al. (2011) also suggested that an influence of TMS on motor programming should have an impact on motor performance in terms of movement accuracy, while an influence on motor attention or intention (Desmurget and Sirigu, 2009) should affect RT (Striemer et al., 2009). However, Snyder et al. (2006) demonstrated in nonhuman primates that RT could be affected when interfering with SPL. Other studies suggested that IPL may also have a role in programming goal directed reaching, whereas the SPL and intraparietal sulcus may be more related to on-line control of movement (Glover, 2004; Pisella et al., 2006).

Thus, our present and previous data (Busan et al., 2009a,c) support the existence of different and partially segregated neural circuits for the implementation of different motor tasks (Jeannerod et al., 1995). They are consistent with the suggestion of a “dorso-medial” stream preferentially involved in reaching movements, classically opposed to a “dorso-lateral” stream that preferably manages reach-to-grasp and/or grasping movements (e.g., Jeannerod et al., 1995; Burnod et al., 1999; Randerath et al., 2010). This suggestion is supported by results obtained with different paradigms and settings (Desmurget et al., 2005; Prado et al., 2005; Fernandez-Ruiz et al., 2007; Filimon et al., 2009; for a review see Vesia et al., 2010).

However, evidence against this possibility has also been advanced (Desmurget et al., 1996; Smeets and Brenner, 1999; Mon-Williams and McIntosh, 2000), showing that the implementation of reaching is not so much segregated, and that wider circuits can participate. In fact, circuits for preparation of reaching may overlap with the neural requests needed for grasping or reach-to-grasp implementation, with still more integrated mechanisms needed for prehension (Binkofski et al., 1998; Smeets and Brenner, 1999; Ulloa and Bullock, 2003; Tunik et al., 2005; Rice et al., 2006). Moreover, it should be noted that regions of the IPL, as for example the angular or the supramarginal gyrus, have been associated with preparation and/or on-line control of visually guided reaching (Koch et al., 2008; Vesia et al., 2008, 2010; Reichenbach et al., 2011).

In the brain, information can be processed serially as well as along parallel pathways (e.g., Burnod et al., 1999; Naranjo et al., 2007; Buneo et al., 2008). Serial organization fits well with the concept that information travels from peripheral to complex “association” areas and then to output channels for action. Similarly, parallel organization of cognitive processes (Cisek and Kalaska, 2010) is supported by a series of studies suggesting that tasks such as visuo-motor integration rely on a network that provides concomitant activation of different cortical regions (Battaglia-Mayer et al., 2006; Naranjo et al., 2007). Our previous data (Busan et al., 2009a,c) support the vision of a mainly serial elaboration of information in this type of task, but also offer a suggestion toward the concept of a parallel elaboration.

TMS/EEG results are in relation with state-dependent activity of the brain, since they were obtained in a resting condition and closed eyes. Brain dynamics were evaluated in a basic condition, usually defined as a “default mode brain state” (Raichle et al., 2001; Raichle and Snyder, 2007; Greicius et al., 2009) where the brain is however active. This reduced the possibility of EEG contamination by movements or other processes.

Concurrent TMS/EEG offers insights into how brain areas interact during information processing (Ilmoniemi and Kicić, 2010). The present work shows propagation of activity ranged mainly from the left somatosensory and parietal structures to right parietal, somatosensory, motor, and more posterior activations. Deep regions of the brain, such as cingulate regions, had significant voxels in late and discrete time windows. Finally, activity in the left temporal and parietal cortices was evident around 190 msec from the delivery of TMS. Instead, when considering the mean neural activity in discrete time of interest, interactions were found among the parietal cortex and, mainly, the posterior regions of the brain. Moreover, significant propagations were found in the left and right frontal regions and in post-central areas, as well as in occipito-temporal regions, and in the right insula.

Our previous studies (Zanon et al., 2010) suggested the presence of an interchange of information between parietal cortex and occipito-temporal cortex at about 170 msec after TMS. An interaction between dorsal and ventral streams has already been proposed (e.g., Himmelbach and Karnath, 2005; Borra et al., 2008; Makuuchi et al., 2012). Valyear and Culham (2010) showed that tool-selective activity was related with parietal and ventral stream activations.

Interestingly, in the present findings, we observed the activation of a neural source around 190 msec from TMS (stimulating the left parietal cortex) in a ventral region in left temporo-parietal cortex. This gives further support to previous results regarding the possibility of an interchange of information between the dorsal and ventral streams: this region has been reported to have a supportive role in the elaboration, for example, of object features in a cross-modal integration (Taylor et al., 2009). Moreover, the analysis of mean neural activity confirmed the presence of neural activations in occipito-temporal regions of the brain in a time window comprised between 130–245 msec, again suggesting interactions between the two systems.

The present findings are in general agreement with previous TMS/EEG studies that assessed the temporal dynamics underlying different tasks. For example, Massimini et al. (2005) investigated how the activation of cortical areas was transmitted to the rest of the brain during wakefulness or sleep. In the case of aware subjects, activation in the parietal cortex was observed about 120 msec after TMS onset. An inverse propagation from the parietal cortex to premotor regions in comparable times was also observed. Another study showed that stimulation of left posterior regions of the brain could elicit activations of frontal areas bilaterally in the first 80 msec from the delivery of the stimulus, depending on a series of variables such as, for example, stimulation intensity or stimulation angle (Casali et al., 2010).

The present findings integrate and extend previous results (Zanon et al., 2010), suggesting the possibility of the existence of wide links between the parietal cortex and other brain regions, ranging from frontal to more posterior parietal, temporal and occipital areas (e.g., Hagmann et al., 2008; Borra and Rockland, 2011). These findings are the result of a series of possible direct and/or indirect links among the highlighted cortical regions. The ones discussed are only the main aspects related to the present pattern of cortico-cortical interactions centered on the parietal cortex, in order to guide the interpretation of behavioral results. Further information about this topic can be found by referring to more specific publications (e.g., Hagmann et al., 2008; Mars et al., 2011).

### Preparation and inhibition of movements

This study shed light on the neural underpinnings of visually-guided reaching movements. The knowledge of regions involved in reaching movement preparation is relevant also to understand the way in which the suppression of reaches is implemented. To this respect it has been proposed that a network composed by the inferior frontal cortex, the subthalamic nucleus and pre-SMA (supplementary motor area) is responsible of inhibitory control (Aron et al., 2007a). In fact, neural substrates of suppression have been found in SMA, pre-SMA, basal ganglia and frontal regions (e.g., Matsuzaka and Tanji, 1996; Aron and Poldrack, 2006; Chen et al., 2010; Mirabella et al., 2012; Swann et al., 2012), as well as in cingulate cortex, insula, prefrontal, fronto-parietal and temporal regions (e.g., Kalaska and Crammond, 1995; Aron et al., 2007b; Chikazoe et al., 2009; Coxon et al., 2009; Stinear et al., 2009; Swick et al., 2011). However, evidence suggests that the motor cortex should be the final target of inhibitory commands that could be elaborated elsewhere (Coxon et al., 2006; Mirabella et al., 2011). Furthermore it has been shown that the parietal cortex can play a role in response stopping or inhibition (e.g., Watanabe et al., 2002; Coxon et al., 2009; Wheaton et al., 2009) and in movement decision-related tasks (Karch et al., 2009). Again these are structures that might also be involved in movement control (e.g., Battaglia-Mayer et al., 2006, 2007; Lindner et al., 2010; Ciavarro and Ambrosini, 2011). Some overlap between inhibition and execution of reaching is witnessed by the fact that strategic changes in movement programming for the very same movements under different cognitive contexts have been shown, requiring different degrees of control during movement (Mirabella et al., 2008). However, Mirabella et al. (2006) showed that the Stop/Go processes interacting in a countermanding task are independent, but likely influenced by a common factor when they are under the control of the same hemisphere.

### Limitations of the study

The present study has a few limitations. The use of slightly different experimental settings could lead to poorly comparable results. However, the lack of significant effects cannot be entirely attributed to the differences adopted. Particular time windows of stimulation or the state-dependent excitability of the cortex could be also critical. Finally, the possibility that different regions could intervene in different manner compared to task requests should be kept in mind.

One of the changes we adopted in the present experiments was to make the subject work with open rather than closed eyes. The rationale for using a “double” RT paradigm in the previous experiments (putatively, a first one from the go signal to the opening of the eyes and a second one from the opening of the eyes to the start of movement; Busan et al., 2009a,b,c) was in relation with the possibility to study a real-time preparation of reaching movements, avoiding that the subject knew in advance positioning of the target. However, it is evident that the presence of a “double” RT represents a complication. On the other hand, in the present experimental setting, a possible effect related to the visual feedback of the arm cannot be completely ruled out. However, in our previous study (Busan et al., 2009c), the evidence of a slower RT only toward the central reaching position could make this point less critical (e.g., Ferraina et al., 1997; Graziano et al., 2000; Buneo and Andersen, 2006; Khan et al., 2007; Filimon et al., 2009; Beurze et al., 2010; Bosco et al., 2010). We should also consider the possibility that different preparations of movement could be present at the same moment in the brain (Cisek and Kalaska, 2010; Cui and Andersen, 2011) before the go-signal, and that subjects simply selected the movement when requested (Cisek and Kalaska, 2010).

We might have not been able to apply TMS at the right time and the possibility that some effects were undetected in present and previous investigations remains, also in relation to the state-dependent theory (Silvanto and Muggleton, 2008). In this sense, the facilitating effects induced by TMS could be also explained as a possible disruption of inhibitory/controlling/competitive processes, which allowed the controlled areas to enhance their functioning (Walsh and Pascual-Leone, 2003).

Specific limitations in the TMS/EEG experiment may also be present. For example, TMS evokes not only responses related to TMS, but also potentials due to acoustic and somatic stimulation. Sham stimulation was implemented to obtain a control for acoustic stimulation, but an optimal control for somatic stimulation is difficult to be obtained. Even if we tried to eliminate the majority of artifacts with ICA (Jung et al., 2000), the possibility remains that these and some other hidden artifacts were still present in the collected data.

## Concluding remarks

The data herein reported contribute to further understand the organization of movements. They are in agreement with the suggestion that SPL is more involved in the preparation of natural reaching compared to more lateral structures. However, TMS/EEG findings showed that parietal cortex stimulation propagates toward a wide system of areas. This suggests that segregation among neural systems is not restrictive, and favors alternative hypotheses suggesting that overlap between different neural structures is needed for the implementation of different movements.

This evidence also represents a complementary point of view with respect to neural organization of movement and response inhibition or stopping, suppression of pending actions, or the quick change of prepared actions. In fact, the organization of reaching and its neural machinery should be highlighted in order to relate them to situations such as inhibition or stopping of action.

### Conflict of interest statement

The authors declare that the research was conducted in the absence of any commercial or financial relationships that could be construed as a potential conflict of interest.
